# Supplementary figures and images for: Framework for rational donor selection in fecal microbiota transplant clinical trials
Source: PLoS One. 2019 Oct 10;14(10):e0222881. doi: 10.1371/journal.pone.0222881 (PMC6786724; doi:10.1371/journal.pone.0222881)

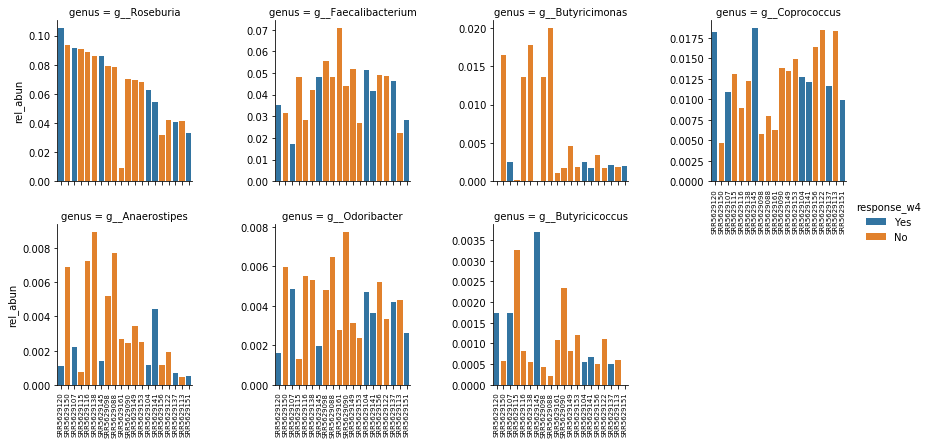

Supplement: S1 Fig — Donors are ordered along the x-axis in the same order for all plots. The respective patient response status is indicated through the bar color (blue = response, orange = no response). (PNG) [file pone.0222881.s001.png]

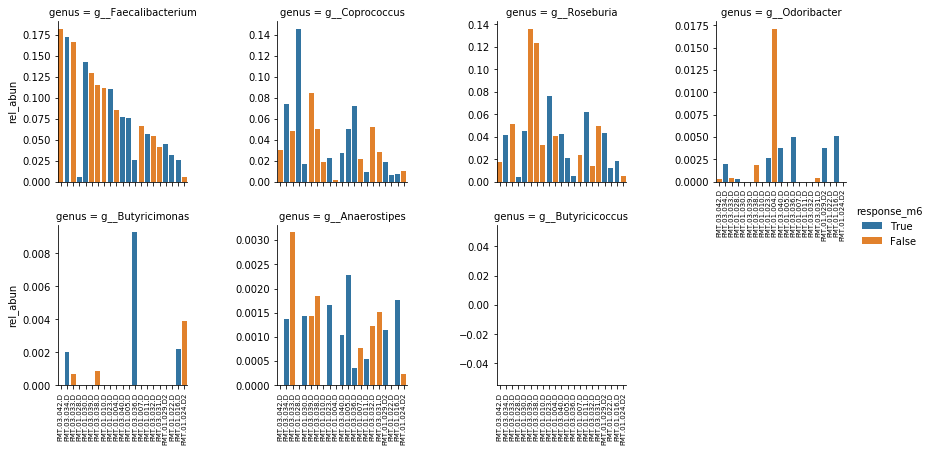

Supplement: S2 Fig — Donors are ordered along the x-axis in the same order for all plots. The respective patient response status is indicated through the bar color (blue = response, orange = no response). Note: Butyricicoccus was not present in any of the donor samples. (PNG) [file pone.0222881.s002.png]

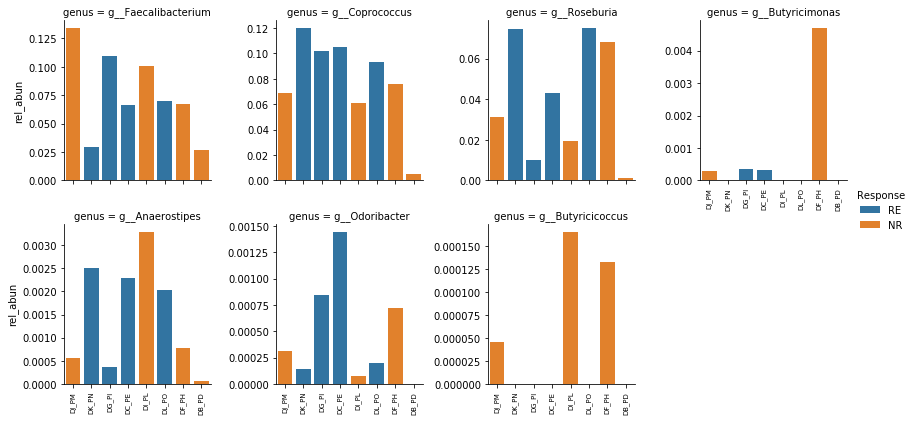

Supplement: S3 Fig — Donors are ordered along the x-axis in the same order for all plots. The respective patient response status is indicated through the bar color (blue = response, orange = no response). (PNG) [file pone.0222881.s003.png]

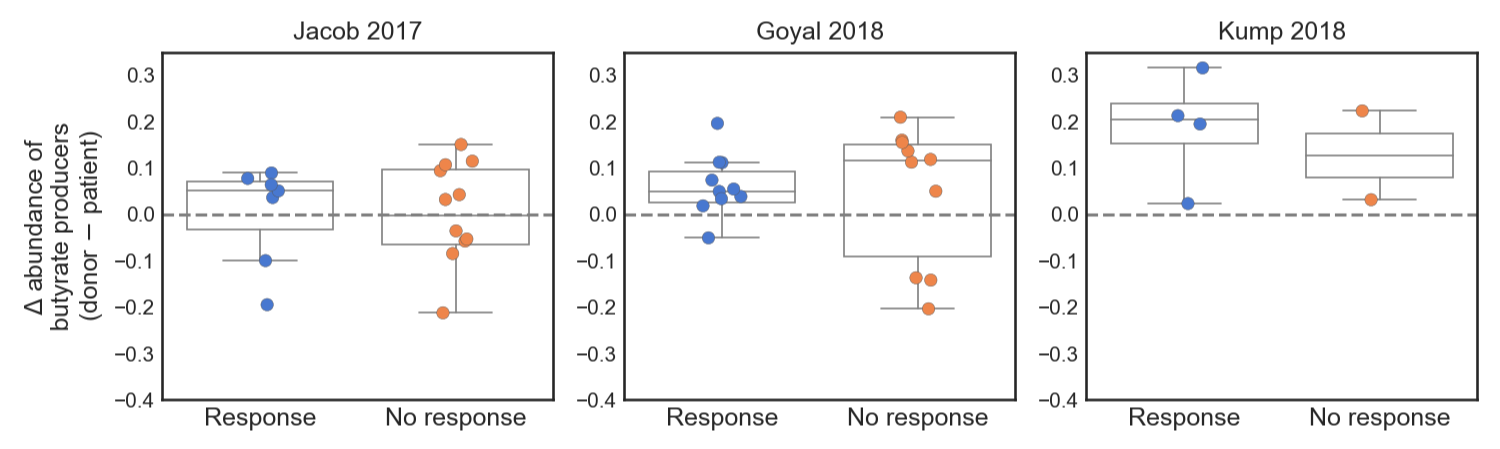

Supplement: S4 Fig — The difference was calculated by subtracting the patient’s total abundance of butyrate producers from the total abundance in their respective donor sample. Butyrate producers were identified as described in the Methods. (PNG) [file pone.0222881.s004.png]
